# Initial institutional experience using a robotic arm–enabled 4K 3D exoscope in neurosurgical operations

**DOI:** 10.3171/2023.10.FOCVID23150

**Published:** 2024-01-01

**Authors:** Jawad M. Khalifeh, Ali Karim Ahmed, Wataru Ishida, Joshua Materi, Anita Kalluri, Daniel Lubelski, Timothy Witham, Nicholas Theodore, Debraj Mukherjee, Judy Huang

**Affiliations:** Department of Neurosurgery, The Johns Hopkins Hospital, Baltimore, Maryland

**Keywords:** robotic exoscope, intraoperative visualization, 3D high-definition visualization, neuronavigation

## Abstract

The extracorporeal telescope (exoscope) presents a novel digital camera system as a versatile alternative to traditional optical microscopy for microsurgery and minimally invasive neurosurgical operations. Recent innovations in exoscope technology offer 4K-definition multiscreen outputs, pneumatic robot arms, 3-dimensional depth perception, and greater illumination, focus, and magnification powers for enhanced intraoperative visualization. The authors present their initial institutional experience using a robotic arm–enabled 4K 3D exoscope in a variety of cranial and spinal neurosurgical operations, namely Chiari decompression, microvascular decompression for trigeminal neuralgia, anterior cervical discectomy, and lumbar decompressions.

The video can be found here:  https://stream.cadmore.media/r10.3171/2023.10.FOCVID23150

## Transcript

In this video, we present our institutional experience using a high-definition 3D exoscope as an alternative to the operating microscope in neurosurgery. Our experience with this particular iteration represents the third of its kind in the United States and the fourth internationally.

### 0:37 Comparison Optical Microscope Versus Exoscope.

The high-powered optical microscope is the gold standard for illumination and magnification in microsurgery.^1^ In this talk, we present our experience using a robotic arm–enabled 4K 3D exoscope.

The exoscope consists of a mobile base on wheels with a robotic arm that attaches to a digital camera.^1^ The camera provides a high-definition image of the field and projects it onto one or multiple 4K-definition high-resolution heads-up display monitors in 2D or 3D stereoscopic visualization.^1,2^ The exoscope offers a larger field of view, greater depth of field, greater illumination and magnification capabilities, as compared to traditional optical microscopy.^2,3^ The exoscope uncouples the surgeon’s body and hands from the traditional microscope eyepiece, thereby offering improved ergonomics and accessibility to the operative field.^4^

Additional features include a tracker pointer instrument with a corresponding pedal, voice-activated commands for intraoperative adjustments in zoom, focus, and a wide range of robotic arm movements.

### 1:38 Initial Case Series.

For the initial implementation, we selected cases based on complexity and ease of integration into the operative workflow. We chose cases that were performed at a high volume, that could be feasibly performed without microscopy, or that could be expeditiously converted to traditional microscope, if needed. We suggest caution regarding initial implementation in cases requiring excessive maneuvering of the scope, or where there needs to be consideration of other large pieces of equipment in the room, such as a surgical robot.

### 2:05 Case Presentation: Chiari Malformation.

We present the case of a 49-year-old woman with a history of strain-induced suboccipital headaches and difficulty with balance and coordination. MRI findings of cerebellar tonsillar descent with Chiari I malformation and syringomyelia.

### 2:19 Operative Steps: Chiari Decompression.

We performed a midline approach for suboccipital craniectomy with C1 laminectomy, under loupes magnification. We use the exoscope for the intradural portions of the operation, which includes intradural exploration with release of arachnoid adhesions, and expansile duraplasty.

### 2:33 Room Setup: Chiari Decompression.

The room setup for a Chiari decompression using the exoscope is similar to what would be used for traditional optical microscopy. The bed is turned 90°, with the operating surgeon and assistant at the head of the bed, the scrub technologist at the foot of the bed, and the anesthetist on the other side. The exoscope base is parked behind the operating surgeon to the left side and the robotic arm comes over the right shoulder of the surgeon, such that the camera overlies the operative field. Two heads-up display monitors are oriented 180° from each other, such that each the primary and assistant surgeon has a view of their monitor over the corresponding surgeon’s shoulder.

As compared to traditional optical microscopy, all participants in the operating room who choose to wear the 3D glasses have a shared 3D stereoscopic view of the surgical field. Shared information among members of the OR care team allows for a greater sense of involvement, efficiency in assistance, educational value, and improved communication.

### 3:29 Operative Videos: Chiari Decompression.

In this recording, the dural opening for Chiari decompression has been completed. We perform arachnoid dissection to facilitate posterior fossa and fourth ventricle outflow exploration. The exoscope affords a wide and well-illuminated view of the operative field. We demonstrate the use of the voice command feature to zoom in, as indicated in the microphone icon on the top right screen.

### 3:54 Operative Videos: Chiari Decompression.

There has been a change in the angle of the exoscope to provide a centered view of the working area. The scope is oriented and focused onto a particular limb of the dural patch that is being sutured. Visualization through the exoscope provides a wide field of view and excellent depth perception for tissue manipulation, dissection, and suturing in a deep operative field.

### 4:18 Case Presentation: Trigeminal Neuralgia.

We present the case of a 77-year-old woman with right-sided sharp, shooting electrical facial pains, consistent with trigeminal neuralgia that is refractory to medical treatment. MRI showed neurovascular conflict with a traversing venous structure abutting the cisternal segment of the right trigeminal nerve.

### 4:36 Operative Steps: Microvascular Decompression.

We performed a right-sided suboccipital retrosigmoid craniectomy, under loupes magnification. Following dural opening, we used the exoscope for the exploration, arachnoid microdissection, inspection of CN V, and bipolar coagulation of the compressive culprit vein.

### 4:52 Operative Steps: Microvascular Decompression.

The MVD operating room setup was similar to that of a Chiari decompression, with the only differences being the positioning of the operating surgeon and assistant at 90° from one another, with corresponding placement of the monitors across from each of them.

### 5:07 Operative Videos: Microvascular Decompression.

The MVD requires camera maneuverability to shifting targets, such as the CN VII/VIII, the petrosal vein, and the trigeminal nerve, within the cerebellopontine angle. We used the exoscope system’s tracked pointer feature. This permits frequent changes to camera angle, magnification, and depth of focus.

Following the dural opening, we identify the tentorium cerebelli and the petrosal vein. Then, we use the tracked pointer to orient the camera along the axis of the pointer and focus deeper within the CP angle to identify the CN V and VII/VIII. From left to right of the screen, the petrosal vein, trigeminal nerve, and facial nerve, all of which are in view. A vein is seen compressing the cisternal portion of the trigeminal nerve. This vein is bipolar coagulated and then cut using microscissors.

### 6:38 Operative Videos: Microvascular Decompression.

Lastly, we use the tracked pointer to adjust the focus of the exoscope at the root entry zone of the trigeminal nerve to examine it along its entire course.

### 7:12 Exoscope Applications Within Spinal Neurosurgery.

The 3D exoscope system lends itself well to applications within spinal neurosurgery.^5,6^ We have used the exoscope in cases of ACDF and lumbar decompressions. The portability of the exoscope permits incorporation in virtually any OR room setup. In these spinal operations, the exoscope base is positioned behind the operating surgeon and the camera is brought overlying the operative field, such that the surgeon and assistant can operate unobstructed, while maintaining an ergonomic upright position.

### 7:41 Case Presentation: Lumbar Disc Herniation.

We present the case of a 43-year-old man with prior left-sided L4–5 discectomy presenting with painful radiculopathy. MRI demonstrated recurrent disc herniation with nerve root compression.

### 7:55 Operative Video: Lumbar Disc Herniation.

We expose the lamina at L4 and L5 and dissect scar tissue from the previous discectomy. The thecal sac and overlying scar tissue are retracted medially to allow for access to the L4–5 disc space. A combination of downgoing curette and pituitary rongeurs are used to free up and resect the large disc herniation, providing decompression of the traversing and exiting nerve roots.

### 8:56 Case Presentation: Lumbar Radiculopathy.

We present another case of a 76-year-old man who presented with persistent left-sided L5 radiculopathy after undergoing a left L4–5 hemilaminectomy. MRI showed stenosis at the left L5–S1 foramen causing compression of the left L5 nerve root.

### 9:16 Operative Video: Lumbar MIS Laminoforaminotomy.

We used a tubular retractor to dock at the level of the left L5 lamina. We used a matchstick drill to remove the inferior left L5 hemilamina and a combination of Kerrison rongeurs to resect the compressive ligamentum flavum and decompress the left L5 nerve root.

### 10:09 Concluding Remarks.

We look forward to exploring applications of the robotic arm–enabled 4K 3D exoscope system in neurosurgical operations, including features to improve intraoperative tissue visualization such as the blue light and indocyanine green filters. We foresee the possibilities of this new tool in improving ergonomics during surgery, visualization of the surgical field, and enhancing neurosurgical education.

A mandatory learning curve should not discourage the adoption of this new technology. We encourage a safe and stepwise escalation in case complexity that builds on an individual surgeon’s comfort level in incorporating changes to their established techniques and workflow.

Thank you for listening.

## Disclosures

Dr. Lubelski reported personal fees from CarboFix, ICOtec, Mindset Medical, and Stryker; and fees to institution (clinical trial) from Dilon Technologies outside the submitted work. Dr. Witham reported being a surgical advisory board member for Augmedics Inc., investment in Augmedics Inc., and being a consultant for Cerapedics and DePuy Synthes Spine outside the submitted work. Dr. Theodore reported royalties from and being a consultant for Globus Medical outside the submitted work. Dr. Huang reported stock ownership in Longeviti outside the submitted work.

## Author Contributions

Primary surgeon: Huang, Lubelski, Witham, Theodore. Assistant surgeon: Khalifeh, Ishida, Materi. Editing and drafting the video and abstract: Huang, Khalifeh, Ahmed, Materi, Theodore, Mukherjee. Critically revising the work: Huang, Khalifeh, Materi, Kalluri, Lubelski, Witham, Theodore, Mukherjee. Reviewed submitted version of the work: Huang, Khalifeh, Materi, Kalluri, Lubelski, Witham, Theodore, Mukherjee. Approved the final version of the work on behalf of all authors: Huang. Supervision: Huang, Khalifeh, Theodore, Mukherjee.

## Supplemental Information

### Patient Informed Consent

The necessary patient informed consent was obtained in this study.
